# Exploring the Experiences of Caring for a Family Member With Intellectual Disabilities Displaying Behaviours That Challenge and/or Mental Health Difficulties Within the United Kingdom: A Meta‐Ethnographic Review

**DOI:** 10.1111/jar.70226

**Published:** 2026-04-17

**Authors:** Lucy Yates, Andrea Flood, Grace Talbot, Alys Wyn Griffiths

**Affiliations:** ^1^ Department of Primary Care and Mental Health University of Liverpool Liverpool UK; ^2^ Division of Neuroscience, School of Medicine and Population Health University of Sheffield Sheffield UK

**Keywords:** challenging behaviour, informal carers, learning disabilities, mental health

## Abstract

**Introduction:**

This review aims to synthesise qualitative literature of experiences of caregiving for family members with intellectual disabilities displaying behaviours that challenge and/or mental health difficulties, within the United Kingdom.

**Method:**

Following Preferred Reporting Items for Systematic reviews and Meta‐Analyses guidelines, five electronic databases (APA PsychInfo, Web of Science, PubMed, MedLine and CINAHL) were systematically searched. Analysis followed a meta‐ethnographic approach.

**Results:**

Fourteen studies were included. Three key themes and a line of argument were developed: (i) Carers Wear Many Hats; (ii) Square Services, Round Needs; (iii) A Journey of Many Lows and Some Highs.

**Conclusions:**

Carers highlighted psychosocial strains of the multi‐faceted caregiving roles—including advocacy, education and protection. One‐size‐fits‐all approaches meant support services were more burdensome than helpful, leaving carers feeling powerless and marginalised, with limited reports of good practice. The findings can inform care services about supporting these families and individuals with idiosyncratic and intersecting needs.

## Lay Summary

- Family members are key caregivers for people with intellectual disabilities who also present with behaviours that challenge and/or mental health difficulties. This review paper highlighted, within the United Kingdom, the various roles and tasks family members take on within their caregiving role, including learning, educating others, navigating and battling with complex care systems and protecting their loved ones.
- Health and social care services were hard to access for this population of individuals, with interventions being offered too late, without enough time to build a relationship and without including family carers. Support was not based on the needs or views of the individual or their family and focused too much on difficulties and problems, without considering progress, strengths, or resources. Professionals sometimes lacked the skills, knowledge and positive attitudes needed when working with this population.
- Taking on caregiving roles negatively impacted on the physical, psychological and social wellbeing of family carers, who often felt isolated and powerless. They also spoke about personal growth, forming new outlooks, experiencing joy and channelling their values and strengths.
- Services must adopt holistic, intensive and person‐centred approaches, which include caregivers and are longer‐term and more proactive. Family carers and professionals working in this field should be provided with regular training and peer/psychological support opportunities.

## 1 Introduction

Approximately 1% of the global population has an intellectual disability (Maulik et al. 2011). Within this population, there are high prevalence rates of mental health difficulties and behaviours that challenge (BtC) (Emerson et al. 2001; Hughes‐McCormack et al. 2017; Pruijssers et al. 2014), which are underreported due to atypical presentations, limited communication and difficulties accessing services (Sheehan et al. 2015). BtC, including self‐injury, withdrawal and destructive behaviours, may serve important functions for the individuals, enabling them to fulfil unmet needs. However, they significantly impact families and care systems around them (Knapp et al. 2005; McIntyre et al. 2002).

Families are key caregivers for people with intellectual disabilities across their lifespan (Lunsky et al. 2014). Parents of children with intellectual disabilities spend the longest time as carers, with 75% caring for over 20 years compared to an average of 18.1% of all carers in England (Emerson et al. 2012). Increasing numbers of adults with intellectual disabilities live with family and friends (Hatton 2017)—the most common permanent residency for this population (Emerson et al. 2012). Caregiving includes support with personal care, supervision for safety and assisting leisure activities (Fraser of Allander Institute 2021). Families are instrumental in organising, liaising with and advocating for support services, especially when relatives displays BtC or mental health difficulties (Weiss and Lunsky 2010).

The experiences of family carers of individuals with intellectual disabilities are well documented, including both ‘joy and sorrow’ (Kearney and Griffin 2001); caregiving can be burdensome, increasing carer stress and poor physical and mental health (Cantwell et al. 2014; Goudie et al. 2014; Marquis et al. 2019). Isolation, unreliable and insufficient care services, reduced employment opportunities and low self‐esteem contribute to this (Marquis et al. 2019).

In the United Kingdom, there has been a drive to keep people with intellectual disabilities out of hospital. Following Winterbourne View (BBC Department of Health 2012), Transforming Care initiated the development of Intensive Support Teams (ISTs) to provide local, intensive and specialist care to individuals with intellectual disability (NHS England). This service reconfiguration placed the management of BtC and mental health difficulties in community‐based facilities, including families (Venville et al. 2015).

People with intellectual disabilities displaying mental health difficulties and/or BtC have several care needs. Challenges associated with providing day‐to‐day care to relatives with intellectual disabilities and BtC include an altered self‐identity and emotional and physical tolls of managing crisis situations (Griffith and Hastings 2014). Fears and low expectations for the future are noted, with a strong motivation—underpinned by love—to obtain excellent support (Griffith and Hastings 2014). Carers described ‘fighting’ for support and reaching ‘crisis’ before it was delivered (James 2013). Services are uncoordinated and bureaucratic, leaving carers at increased risk of psychosocial distress (James 2013).

The latest reviews within this area (Griffith and Hastings 2014; James 2013) were conducted over a decade ago. Little is known about how service changes have impacted on family members' experiences of providing care. The current review aimed to explore experiences of family carers of individuals with intellectual disabilities and BtC and/or mental health difficulties within the United Kingdom.

## 2 Methods

The review protocol was registered on PROSPERO (ref: CRD42023472275). It followed Preferred Reported Items and Meta‐Analysis (PRISMA) guidelines (Page et al. 2021) and the seven steps of meta‐ethnography (Noblit and Hare 1988; Sattar et al. 2021).

### 2.1 Positionality and Reflexivity

The authors are clinical psychologists. researchers, and an assistant psychologist who bring together relevant professional experience of conducting research into and working clinically with marginalised populations, including within learning disability and intensive support services. They have lived experiences of directly and indirectly caring for family members with additional needs, including learning disabilities. Throughout the research process, the authors kept reflexive journals and frequently discussed how their experiences and preconceived beliefs may impact the research process and interpretation. Steps were taken to mitigate this; for example, clinician–researchers must identify the risk of being overly critical of their profession (Hiller and Vears 2016); therefore, the authors were careful to avoid aspects being silenced or aggrandised.

### 2.2 Search Strategy

Scoping searches were run in November 2023. Systematic searches were run in May 2024, in APA PsycInfo, Web of Science, PubMed, MedLine and CINAHL. Hand searches on Google Scholar and forward and backward citation chaining were utilised.

Search terms were formulated by Authors L.Y. and A.F., based on existing literature with librarian consultation. Key search concepts were: ‘Intellectual Disabilities’, ‘Mental Health’, ‘Behaviours that challenge’, ‘Family carers’ and ‘Experiences', combined with the ‘AND’ Boolean operator. These were expanded to form the full search strategy (see Supporting Information).

### 2.3 Eligibility Criteria

Family carers included unpaid or informal carers of individuals (of any age) with intellectual disabilities; formal and paid carers were excluded. Articles focused specifically on carers' experiences of their family members' BtC and/or mental health.

Articles were included if participants' relatives had other diagnoses/difficulties co‐occurring with intellectual disabilities. Articles were excluded if they primarily focused on individuals with other diagnoses/difficulties, whereby an intellectual disability was not compulsory for participation. The review aimed to update the Griffith and Hastings (2014) review, which included articles published up to and including 2011; we only included peer‐reviewed literature dated from 2012 onwards. Papers utilising qualitative or mixed methodologies whereby qualitative data could be extracted were deemed suitable for understanding caregivers' experiences and were included. Papers were required to be UK‐based, therefore, papers conducted outside the United Kingdom were excluded.

Authors L.Y. and G.T. independently screened all titles and abstracts. Full‐text papers of titles/abstracts were independently assessed by both researchers against inclusion criteria. Discrepancies were resolved by consensus through discussion with Authors A.F. and A.W.G.

### 2.4 Study Selection

**FIGURE 1:**
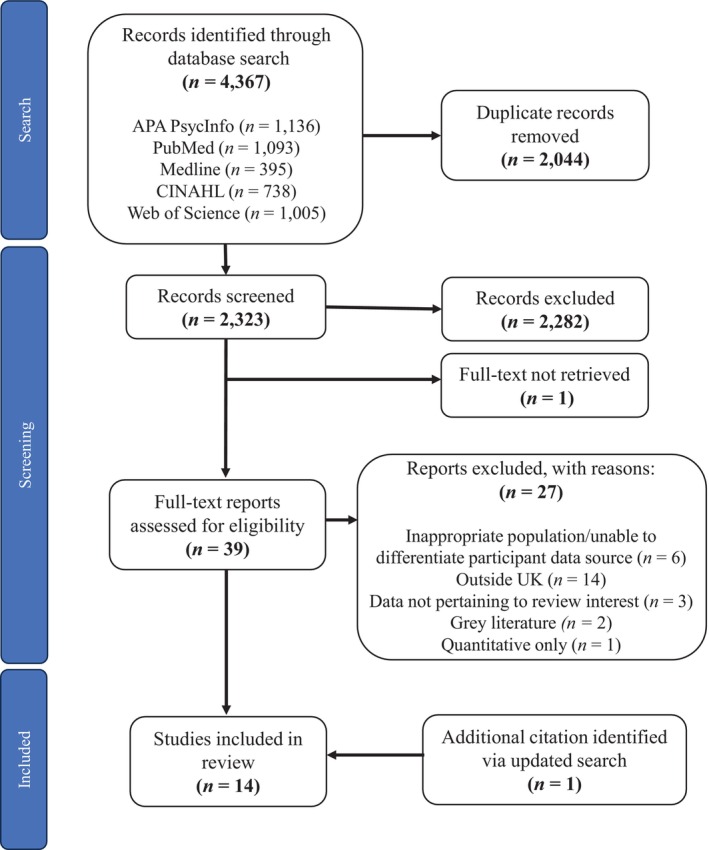
PRISMA diagram for searches.

The search yielded 4367 reports. Following duplicate removal, 2323 were screened for inclusion (Figure 1). Screening titles and abstracts resulted in 40 potential articles, of which 39 full texts were obtained, with Hughes et al. (2024) unavailable in full text. Following full text screening, fourteen articles were included in the review.

### 2.5 Quality Assessment

The Critical Appraisal Skills Programme (2018) guided evaluation of the articles' usefulness, validity and rigour (Boland et al. 2017). Papers were assessed independently by two authors (Author L.Y. and A.W.G.), resolving discrepancies through discussion. Scores were assigned to each question, establishing if the criteria had been met entirely (1), partially/not possible to tell (0.5), or not at all ([0]; Butler et al. 2016). This contributed to overall scores, which were classified as high‐quality (9–10), moderate‐quality (7–5–8.5) or low‐quality ([< 7.5]; Butler et al. 2016).

### 2.6 Data Extraction and Synthesis

To describe the review, the following data was extracted from each article: author(s), publication year, focus/aims, participants, recruitment/setting and data collection and analysis methods (see Table 1). Data was extracted from ‘results’, ‘findings’ and ‘discussions’, including raw data, author interpretations, conclusions and clinical implications.

**TABLE 1:** Study characteristics.

Author (year)	Focus	Participants	Setting/sampling	Data collection	Data analysis
Jacobs et al. (2016)	Parents understanding of causes for their child's BtC, focused on locus of control and stability	*n* = 10 9 mothers, 1 father	Recruited through specialist education schools and voluntary organisations	Individual semi‐structured interviews utilising vignettes	Thematic analysis
Ross and Dodds (2023)	Risk factors for admission to inpatient intellectual disability services	*n* = 7 5 relatives 2 young people	Single child and adolescent mental health service (CAMHS) inpatient ward	Individual semi‐structured interviews	Interpretive phenomenological analysis
James (2016)	Family carers experience of their relative's admission to an Assessment and Treatment Unit (ATU)	*n* = 6 3 mothers, 3 fathers (including 1 stepfather)	Three ATUs	Individual semi‐structured interviews	Interpretive phenomenological analysis
Sheehan et al. (2018)	Family carers views on the management of BtC, focusing on psychotropic medication	*n* = 99 family carers	Recruited via social media and voluntary organisations	Online survey	Descriptive analysis
Botterill et al. (2019)	Family members' perspectives of positive behaviour support (PBS)	*n* = 8 6 mothers, 2 fathers	Two community teams providing PBS to young people with intellectual disabilities	Individual semi‐structured interviews	Inductive thematic analysis
Adams and Jahoda (2019)	Mothers' experiences of seeking support for their offspring with severe/profound intellectual disabilities and emotional difficulties	*n* = 7 mothers	Recruited via voluntary organisations	Individual semi‐structured interviews	Interpretive phenomenological analysis
Gore, McGill, and Hastings (2019)	Experiences of caregiver's goal selection within PBS and the psychological and emotional processes involved	*n* = 12 8 mothers, 1 father, 3 grandparents	Two CAMHS CLDTs	Individual semi‐structured interviews	Framework approach
Kiernan et al. (2019)	Mother's experiences of children with intellectual disabilities and behavioural needs	*n* = 10 mothers	Recruited via a network supporting families of individuals with intellectual disabilities	Individual semi‐structured interviews.	Phenomenological thematic analysis
Kouroupa, Hamza, et al. (2023)	Stakeholders' perspectives on ISTs	*n* = 50 Family carers (*n* = 9; 7 parents, 1 brother, 1 aunt) Paid carers (*n* = 7) Individuals with intellectual disabilities (*n* = 6; age range 26–40 years) IST professionals (*n* = 28)	Seven ISTs	Individual semi‐structured interviews.	Thematic analysis
Kouroupa, Hassiotis, et al. (2023)	Facilitators and barriers to reducing aggressive BtC for adults with mild/moderate intellectual disabilities	*n* = 40 Family carers *n* = 7 14 triads of individual with intellectual disability; family or paid carer; health or social care professional involved in their care	Seven CLDTs	Semi‐structured interviews with triads of participants.	Thematic analysis
Chase and McGill (2019)	Adult siblings' perspectives of individuals with intellectual disabilities and BtC	*n* = 6 female siblings	Recruited via voluntary organisations	Individual semi‐structured interviews.	Interpretive phenomenological analysis
McKenzie et al. (2018)	Family carers experiences and conceptualisation of PBS	*n* = 8 family members	Recruited via voluntary organisations	Individual interviews (*N* = 3) Focus group (*N* = 5)	Inductive thematic analysis
Paulauskaite et al. (2021)	Impact of coronavirus on families of preschool children with moderate/severe intellectual disability and BtC	*n* = 88 parents (84 females, 4 males)	Recruited from email list of a larger clinical trial	Online survey	Inductive content analysis
Stock et al. (2024)	Family experiences of hospitalisation and discharge for individuals with severe intellectual disabilities and complex needs	*n* = 6 4 mothers, 1 father, 1 sister	Recruited via voluntary organisation and social media	Focus groups	Inductive reflexive thematic analysis

A meta‐ethnographic approach was used (Boland et al. 2017; Noblit and Hare 1988) and expressed following reporting guidance (France et al. 2019). First‐order constructs (e.g., participant quotes) were re‐interpreted alongside second‐order constructs (e.g., thematic concepts) while preserving their contextual information (Boland et al. 2017). As papers were coded, new themes were developed and existing themes were added to. Papers were translated into one another, using *reciprocal* and *refutational translation* to note similarities and differences across articles. Through an iterative process, moving back and forth between the extracted data and identified themes, third‐order themes were developed while acknowledging gaps in the data. A *line‐of‐argument* synthesis was established from third‐order constructs (Noblit and Hare 1988).

## 3 Results

### 3.1 Quality Assessment

The quality assessment yielded scores ranging from 5.5 to 9 (see Table 2). Three papers were rated as high‐quality, eight as moderate‐quality and three as low‐quality (Butler et al. 2016). No papers were excluded based on quality; however, higher quality papers were utilised more during synthesis. If only lower quality papers contributed to themes, these were refined and integrated into other themes (Siddaway et al. 2019).

**TABLE 2:** Overview of quality assessment. Scoring key: 1 = Yes, 0.5 = Partially, 0 = No/Can't tell.

	Paper	1. Clear statement of aims?	2. Qualitative methodology appropriate?	3. Research design appropriate?	4. Recruitment strategy appropriate?	5. Was data collected in a way to address research question?	6. Was the relationship between researcher and participant adequately considered?	7. Ethical issues considered?	8. Data rigorously analysed?	9. Clear statement of findings?	10. How valuable is the research?	Overall score^a^
1	Jacobs et al. (2016)	1	1	1	0.5	1	0	0.5	1	1	1	8
2	Ross and Dodds (2023)	1	1	1	1	1	0	1	1	1	1	9
3	James (2016)	1	1	1	0.5	1	0	0	1	1	1	8.5
4	Sheehan et al. (2018)	0	1	0.5	1	0.5	0	0.5	0.5	1	1	6
5	Botterill et al. (2019)	1	1	1	0.5	1	0	0	1	1	1	7.5
6	Adams and Jahoda (2019)	1	1	1	1	1	0.5	0.5	1	1	1	9
7	Gore, McGill, and Hastings (2019)	1	1	1	0.5	1	0	0	1	1	0.5	7
8	Kiernan et al. (2019)	1	1	1	1	1	0	0	1	1	1	8
9	Kouroupa, Hamza, et al. (2023)	0.5	1	1	1	1	0	0	1	1	1	7.5
10	Kouroupa, Hassiotis, et al. (2023)	1	1	1	1	1	0	0.5	1	1	1	8.5
11	Chase and McGill (2019)	1	1	1	1	1	0	0	1	1	1	8
12	McKenzie et al. (2018)	1	1	1	1	1	0	0	1	1	1	8
13	Paulauskaite et al. (2021)	0.5	1	0.5	1	0.5	0	0	0.5	1	0.5	5.5
14	Stock et al. (2024)	1	1	1	1	1	0.5	0.5	1	1	1	9

^a^ Sum of checklist item scores (10 is maximum).

All papers used appropriate methodologies and provided clear statements of findings. However, there was limited consideration of ethical issues and the relationships between researchers and participants. Three papers (Kiernan et al. 2019; Kouroupa, Hassiotis, et al. 2023; McKenzie et al. 2018) included limited relevant qualitative data that could be synthesised.

### 3.2 Participant Characteristics

Across all papers, the views of 281 relatives of individuals with intellectual disabilities were obtained. Eleven studies included the views of relatives, three included the views of individuals with intellectual disabilities alongside relatives (Ross and Dodds 2023), of which two also included paid carers and/or healthcare professionals (Kouroupa, Hamza, et al. 2023; Kouroupa, Hassiotis, et al. 2023). Only data explicitly generated by family carers were extracted; secondary data was not coded or synthesised when it was not possible to distinguish who contributed the data.

Six papers were parents only (Adams and Jahoda 2019; Botterill et al. 2019; Jacobs et al. 2016; James et al. 2020; Kiernan et al. 2019; Paulauskaite et al. 2021), one was parents and a sibling (Stock et al. 2024), one was siblings only (Chase and McGill 2019) and two included parents and extended family members (Gore, McGill, and Hastings 2019; Kouroupa, Hamza, et al. 2023). The remainder did not clarify the nature of the familial relationships.

From the eight studies that included the data, there was a pooled total of 150 parents, with 136 mothers and 14 fathers, including one stepfather. There were three grandparents, one aunt and 80 siblings (seven females, one male). Five papers described the ages of carers (range = 22–70 years).

### 3.3 Characteristics of Family Members With Intellectual Disabilities

Family members with intellectual disabilities were under 18 years old in five studies, with a range of 2.5–18 years (Botterill et al. 2019; Gore, McGill, and Hastings 2019; Jacobs et al. 2016; Kiernan et al. 2019; Ross and Dodds 2023); adults in six studies reported ages ranging from 18 to 50 (Adams and Jahoda 2019; Chase and McGill 2019; McKenzie et al. 2018; Sheehan et al. 2018; Stock et al. 2024); and were unspecified in the remaining papers. Based on available data in six articles, family members with intellectual disabilities included 44 females and 120 males. Other demographic information about carers and their family members was inconsistently reported.

### 3.4 Design Characteristics

All papers were UK‐based and published after the national service model for transforming care services was published (NHS England 2015), though one collected data from 2009 to 2013 (James 2016). Two were conducted in Scotland (Adams and Jahoda 2019; Jacobs et al. 2016), one in Wales (James 2016), two in the North of England (McKenzie et al. 2018; Ross and Dodds 2023), one in the South of England (Gore, McGill, and Hastings 2019), two recruited from across England (Kiernan et al. 2019; Paulauskaite et al. 2021) and one recruited from across the United Kingdom (Kouroupa, Hassiotis, et al. 2023). The rest did not detail recruitment locations.

Recruitment methods included purposive sampling from relevant services and organisations in 13 papers; participants in 5 of these papers were approached by researchers, were self‐selecting in 4 papers, a combination of both in 1 paper and unable to distinguish in 3 papers. In one paper, participants were self‐selecting via an online survey.

Four studies explored carers' experiences and understandings of BtC (Chase and McGill 2019; Jacobs et al. 2016; Kiernan et al. 2019), with one focusing on the impact of COVID‐19 (Paulauskaite et al. 2021). Three papers explored experiences of mental health admissions (James 2016; Ross and Dodds 2023; Stock et al. 2024), one looked at support‐seeking for mental health difficulties (Adams and Jahoda 2019) and one explored stakeholders' perspectives of ISTs (Kouroupa, Hamza, et al. 2023). One paper focused on psychotropic medication for managing BtC (Sheehan et al. 2018) and one explored barriers and facilitators of reducing aggressive BtC (Kouroupa, Hassiotis, et al. 2023). Three papers examined perspectives of positive behaviour support (PBS), an approach for understanding and supporting with BtC (Botterill et al. 2019; Gore, McGill, and Hastings 2019; McKenzie et al. 2018).

One study conducted interviews with triads, including individuals with intellectual disabilities, a family or paid carer and a health or social care professional (Kouroupa, Hassiotis, et al. 2023). One study used vignettes alongside semi‐structured interviews (Jacobs et al. 2016). Two papers used online surveys (Paulauskaite et al. 2021; Sheehan et al. 2018), one employed focus groups (Stock et al. 2024) and one utilised focus groups and individual interviews (McKenzie et al. 2018). The remainder collected data via individual semi‐structured interviews only.

### 3.5 Meta‐Ethnography Findings

Three main themes were developed: (i) Carers Wear Many Hats; (ii) Square Services, Round Needs and (iii) A Journey of Many Lows and Some Highs (see Table 3).

**TABLE 3:** Summary of themes.

Master theme	Subtheme	Contributing articles
Carers wear many hats	Identity and responsibility as a carer	1, 3, 5, 6, 9, 11, 12, 14
	A learning process	1, 2, 3, 5, 6, 7, 8, 11, 13
	Educating others	1, 6, 8, 10, 12, 14
	Advocacy, fighting and protection	2, 3, 8, 9, 10, 12, 14
Square services, round needs	‘Deep‐rooted systemic issues’	2, 3, 6, 9, 10, 12, 13, 14
	Poor attitudes and a lack of competence	4, 6, 8, 12, 14
	‘One‐size‐fits‐all’ and problem‐saturated approaches	4, 5, 6, 7, 8, 10, 12, 14
	The positive difference of an accessible and knowledgeable professional	3, 4, 5, 6, 9, 10, 12, 14
A journey of many lows and some highs	‘End of my tether’	2, 3, 5, 6, 7, 10, 11, 12, 14
	Powerless and undervalued	1, 3, 4, 5, 6, 8, 12, 14
	Isolation and exclusion	2, 3, 4, 5, 7, 8, 10, 11, 12, 13, 14
	A positive shift	3, 5, 7, 8, 11, 14

#### 3.5.1 Carers Wear Many Hats

This theme highlights the high levels of demands and multifaceted roles of caregiving.

##### 3.5.1.1 Identity and Responsibility as a Carer

Being a carer was a defining feature of participants' identities and sense of selves, with their lives ‘revolving’ around the person they cared for (Kiernan et al. 2019). Similarly, carers played a central role in their relatives' lives—ensuring their safety, wellbeing and service access (McKenzie et al. 2018)—both carers of adults (McKenzie et al. 2018) and children (Jacobs et al. 2016).

During admissions, carers felt ‘insecure’ about their role because professionals took over their relative's care (James 2016). Carers ‘justified’ the need for admissions—discussing the complexity of needs and diagnoses, possibly to psychologically defend against role insecurity. Siblings adopted parentified roles—‘like he's my child […] a lot of responsibility for his well‐being’ (Chase and McGill 2019, 142).

Carers rarely spoke about love; however, it did feature in authors' interpretations (McKenzie et al. 2018). Caregiving was underpinned by wanting the best for their relatives; wanting them to be happy, treated with respect and dignity, kept safe and given choices and access to opportunities (McKenzie et al. 2018). This was depicted by relatives being the predominant focus of carers' stories (Stock et al. 2024). While caring responsibilities were assumed ‘willingly’, carers knew there was no‐one else to do it and/or their relatives' quality of life would be poorer if they abstained (Chase and McGill 2019). Siblings felt pressure to relieve parents of caring responsibilities (Chase and McGill 2019).

##### 3.5.1.2 A Learning Process

Carers were ‘learning all the time’ (Jacobs et al. 2016, 64) about their relatives' diagnoses, behaviours and needs; strategies for managing BtC; and how to navigate complex systems. Carers viewed themselves as ‘experts’ of their relative over professionals ‐ ‘we knew him best’ (McKenzie et al. 2018, 61). However, emotional difficulties manifested in ways difficult for carers to understand (Adams and Jahoda 2019), which was especially challenging when relatives with intellectual disabilities lacked insight into, understanding of or ability to communicate their distress (Kiernan et al. 2019; Ross and Dodds 2023).

Acquiring knowledge enabled carers to become ‘parenting professionals’ (Kiernan et al. 2019, 179), potentially increasing their power when liaising with services. Opportunities to develop understandings of loved ones' behaviours seemed to foster hope, empathy and helped carers feel more in control and confident to manage BtC (Botterill et al. 2019).

##### 3.5.1.3 Educating Others

Carers adopted educative roles. They helped their relatives to understand their own BtC and taught them more adaptive behaviours (Jacobs et al. 2016). Carers shared knowledge via peer support groups and informed the creation of specialist provisions (Kiernan et al. 2019).

Authors less explicitly identified carers' roles of educating professionals, despite viewing themselves as experts. Carers wanted professionals to value and collaboratively utilise their in‐depth ‘grounded knowledge’ (James 2016, 45). They ‘compiled detailed documentation’ and made ‘communication boards for the staff’ to facilitate understanding of their relatives during admissions (Stock et al. 2024). In PBS interventions, carers offered successful suggestions to care providers due to informally ‘“doing” PBS long before professionals’ (McKenzie et al. 2018, 61).

##### 3.5.1.4 Advocacy, Fighting and Protection

Carers took on advocacy roles to enable their relatives to access services and opportunities. These experiences were described in emotive and adversarial ways, using words like ‘battle’ and ‘fight’ and being ‘under attack’. Some recalled ‘over 20 years of arguing with and challenging health and social care’ (McKenzie et al. 2018, 60). Others ‘had to’ become ‘a pain in the arse’ (Stock et al. 2024) and ‘exert their authority’ to be heard (James 2016, 47), suggesting they had no choice in the matter.

Carers possibly experienced a sense of push‐pull; while they wanted their loved ones to have equal access to opportunities, they felt protective of—and proactively removed—them from opportunities to prevent bullying and formal exclusion (Ross and Dodds 2023; Kiernan et al. 2019). In inpatient settings, carers wanted to protect their loved ones who were experiencing ‘abusive practice’ from staff (Stock et al. 2024, 7), though worried about potential ‘backlash’ for relatives when raising issues (McKenzie et al. 2018). There was also a need for carer self‐preservation (James 2016).

#### 3.5.2 Square Services, Round Needs

This theme captures carers predominantly ‘dissatisfying’ experiences of support services, interventions and professionals.

##### 3.5.2.1 Deep‐Rooted Systemic Issues

The complexity of accessing and navigating services was clear, with a significant shortage of support options, especially for those with severe or profound intellectual disabilities (Adams and Jahoda 2019). ‘Inappropriate placements’ and being ‘let down’ by health and social care resulted in ‘locking people unnecessarily’ in hospitals—described as ‘prisons’—resulting in severe escalations in BtC and relatives being ‘traumatised’ (Stock et al. 2024, 5).

Mainstream mental health services were unable to adapt interventions or did not accept individuals with intellectual disabilities (Kouroupa, Hassiotis, et al. 2023). Respite provisions could not safely manage the complexity of intersecting needs and risks (Ross and Dodds 2023). While some experienced ISTs helping them access other services (Kouroupa, Hamza, et al. 2023), requests for help being ignored until professionals advocated for them was a barrier (Botterill et al. 2019). Carers described disappointment when ‘promises’ of support accounted to nothing (Kouroupa, Hassiotis, et al. 2023)—feeling ‘lied to’ and ‘scammed’ (Stock et al. 2024, 7).

Help was reactive; families reached ‘crisis point’ before they could access support (Adams and Jahoda 2019). Even then, support was slow with families waiting ‘4 months for a hospital bed’ (Ross and Dodds 2023, 290). Delays contributed to their relatives' mental health deteriorating and consequently longer admissions and increased restraints (Ross and Dodds 2023). ‘Harrowing’ experiences of hospitalisation meant carers went from ‘begging’ for admissions to ‘begging’ solicitors to help them appeal the admission; the relief of their relative's discharge felt like ‘winning the 100 million Euro lotteries’ (Stock et al. 2024, 5).

Poor co‐ordination left services ‘battling’ among themselves (Ross and Dodds 2023, 288), contributing to inconsistent care—alongside high staff turnover (Ross and Dodds 2023), long waiting lists and ‘service gaps’ (Kouroupa, Hassiotis, et al. 2023). Carers possibly felt hopeless, constantly being ‘back to square one’ with seeing different professionals (Ross and Dodds 2023, 288).

##### 3.5.2.2 Poor Attitudes and a Lack of Competence

Carers highlighted professionals' low levels of willingness, pessimism and stereotypical and discriminatory views (Adams and Jahoda 2019). Relatives in hospital were ‘not treated like human beings’ (Stock et al. 2024, 5). Others were metaphorically ‘put in the rubbish bin’ (Adams and Jahoda 2019, 139), with professionals underestimating their abilities and potential to benefit from therapy. Professionals seemingly had ‘hidden agendas’ to ‘section’ one relative, that is, detain them under the (Mental Health Act 2007; McKenzie et al. 2018).

Carers were disheartened and frustrated by professionals' limited specialist knowledge and skills. One carer recalled a doctor with ‘no experience of special educational needs’ (Sheehan et al. 2018, 89), while others lacked essential communication training to interact with relatives (Stock et al. 2024). Unhelpful experiences of care led to distrust and reduced confidence in services and reluctance to seek help (Adams and Jahoda 2019). Within residential settings, there was a sense of relief when staff turnover meant ‘negative’ staff left as they were ‘fuelling challenging behaviour’ (McKenzie et al. 2018, 59).

Practitioners' limited training opportunities, over‐stretched resources and their own feelings of ‘powerlessness’ within ‘bureaucratic systems’ may partly explain these experiences (Kiernan et al. 2019; McKenzie et al. 2018).

##### 3.5.2.3 ‘One‐Size‐Fits‐All’ and Problem‐Saturated Approaches

Carers experienced interventions that were not person‐centred and there were conflicting carer‐professional expectations about appropriate support (Kouroupa, Hassiotis, et al. 2023). Relatives were ‘drugged up’, sometimes on medications they did not have appropriate diagnoses for (Stock et al. 2024). Interventions were offered in ‘short bursts’ when things ‘got tough’ (Kouroupa, Hassiotis, et al. 2023). Longer interventions were needed to enable their relatives ‘to build confidence in people’ and for professionals to understand distinctive communications (Adams and Jahoda 2019, 140). Including carers in psychotherapeutic interventions meant they could ‘break it down’ into the ‘kind of language’ that works for their relative (Kouroupa, Hassiotis, et al. 2023, 10). ‘On‐going support’ was wanted given the ever‐evolving nature of the ‘problems’ (Botterill et al. 2019, 97), with discharge not being ‘the end of the road’ (Stock et al. 2024, 8). It was imperative for interventions to be meaningfully tailored to the family's resources, preferences, strengths and goals to prevent carers feeling overwhelmed and demotivated (Botterill et al. 2019).

Carers were reluctant for professionals to focus solely on BtC and problem‐saturated narratives, wanting recognition of strengths, skills and progress—to ‘enjoy something positive’ (Botterill et al. 2019, 96). Experiencing this helped re‐energise them, instilled hope and increased their ‘joy and enthusiasm’ (Gore, McGill, and Hastings 2019, 1708).

##### 3.5.2.4 The Positive Difference of an Accessible and Knowledgeable Professional

Positive experiences of care were apparent, mostly in relation to individual professionals and specific interventions. Having an accessible point of contact was helpful (James 2016). Carers valued relationships with professionals based on ‘honesty’, ‘genuine interest’, ‘collaboration’ and being ‘listened to’. These experiences motivated carers to attend sessions and adhere to care plans (Kouroupa, Hassiotis, et al. 2023). It seemed, for some, positive experiences were based on being treated with basic respect and having their fundamental rights preserved; ‘at least now they're listening to us’ (James 2016, 48).

Some spoke positively of professionals delivering PBS, emphasising their knowledge and skills, patience in implementing and modifying plans and giving advice in non‐blaming ways (Botterill et al. 2019). Holistic approaches, including carer‐specific emotional support (Botterill et al. 2019) and experiences of ‘hugely intensive’ support—where practitioners spent significant time with families—were ‘phenomenally beneficial’ (Kiernan et al. 2019, 181). This could reduce carers' feelings of isolation and increase their confidence in interventions and services. It seemed empowering when professionals valued carers' input and provided them with information to help understand their relatives better. Voluntary sector organisations were a ‘lifeline’ (Adams and Jahoda 2019).

#### 3.5.3 A Journey of Many Lows and Some Highs

This theme captures the detrimental—and less frequent beneficial—psychosocial impacts of caregiving.

##### 3.5.3.1 End of My Tether

Caregivers spoke about the significant, ‘far‐reaching’ and painful experiences of caring, using language like ‘overwhelming sense of failure’, ‘heartbreak’, ‘guilt’, ‘blame’, ‘bitterness’, ‘loss of confidence’ and ‘regret’. Sadness was common and seemingly never‐ending, depicted by a carer who ‘just cried and cried and cried and cried and cried and cried’ (McKenzie et al. 2018, 61). Carers of individuals in hospital experienced a ‘horrendous toll’ on their mental and physical health, describing suicidal ideation and high blood pressure (Stock et al. 2024, 6).

There was a sense of fragility in carers' perceived abilities to cope, especially when caring for children and looking to the future (Jacobs et al. 2016). The future felt uncertain and daunting, knowing support would always be needed (Stock et al. 2024). Carers of adult‐children perceived that living with BtC for ‘nearly 40 years […] doesn't make it easier’ (McKenzie et al. 2018, 61).

The physical and psychological impacts of experiencing threats to their own safety with aggressive BtC went unspoken. One carer described not wanting to be around her relative, which was a ‘horrible feeling […] made me feel awful and I certainly haven't said it to many people’ (Gore, McGill, and Hastings 2019, 1709). Carers possibly feel ashamed about—and are reluctant to discuss—these painful thoughts and feelings. Carers centred their stories on their relatives' ‘dire’ experiences, considering their own pain as secondary (Stock et al. 2024).

For most, emotional challenges were compounded by limited accessible and specialist support for themselves (Kouroupa, Hassiotis, et al. 2023), especially for sibling carers who were not ‘accounted for’ in the same way as parents (Chase and McGill 2019).

##### 3.5.3.2 Powerless and Undervalued

Carers experienced pervasive feelings of powerlessness. Some were unable to understand and help their distressed relatives (Adams and Jahoda 2019). Carers felt powerless in crisis situations—‘we had to ring the police […] there were times he was becoming extremely violent’ (Ross and Dodds 2023, 287). PBS approaches put ‘control back’ to families, suggesting control had been lost (Botterill et al. 2019).

However, experiences of powerlessness predominantly resulted from being under the ‘power and control of “the system”’ (McKenzie et al. 2018, 60). Disempowerment was an active process by professionals who were ‘pulling rank’ and treating carers as ‘second class’ (McKenzie et al. 2018). One consultant was depicted as the ‘king of the castle’ while carers were ‘not important’ as they were ‘only the parents’ (James 2016, 43). It was unclear if this was an internalised narrative or their perception of how others viewed them (James 2016). Carers felt ‘helpless’ in treatment decisions (Sheehan et al. 2018), reflected by their concerns only being taken seriously when professionals advocated for them (Botterill et al. 2019). On one hand, carers felt powerless due to professionals being gatekeepers and declining support requests. On the other hand, carers were ‘subjugated’ and ‘devalued’ by professionals who ‘take over’ the care of their relative (James 2016). There was a perceived need to comply with services, ‘abide by the rules’ and reluctantly accept inappropriate care (James 2016, 43). This was partly to avoid ‘alienation’ (Kiernan et al. 2019) and to ‘keep the peace’ to prevent jeopardising placements (Stock et al. 2024).

##### 3.5.3.3 Isolation and Exclusion

Closely interlinked to carers' experiences of powerlessness were paralleled experiences of exclusion, isolation and marginalisation for themselves and their relatives (Stock et al. 2024). It seemed there was ‘nobody there to help’ (Adams and Jahoda 2019, 139). Even when support was offered, treatment decisions were made without involvement of carers, who were ‘completely ignored’ (James 2016, 42). It is possible experiences of ‘battling’ against services heightened carers' isolation due to them actively working against one another.

Authors described mothers' anger and disappointment at the health inequalities and discrimination by care services that their offspring faced due to their intellectual disability (Adams and Jahoda 2019). Individuals were expected to change to ‘fit in’ rather than services adapting (Gore, McGill, and Hastings 2019, 1706). They were excluded from community clubs, mainstream services and respite provisions because of their behaviours and needs not fitting inflexible eligibility criteria—increasing carer burden and contributing to relatives becoming ‘more depressed’ and ultimately needing admissions (Ross and Dodds 2023).

Carers felt isolated from peers due to limited opportunities (Kiernan et al. 2019) and others not understanding their experiences (Stock et al. 2024). Some were not working due to caregiving responsibilities (Kiernan et al. 2019). One carer described the ‘massive, massive loss’ of their family never going out together because of their child's behaviours (Gore, McGill, and Hastings 2019). There were varied experiences of relationships within families; some became closer and some more distant and even hostile due to different responses to their situation (Chase and McGill 2019). Relatives ‘lost trust’ in their carers following admissions (Stock et al. 2024).

Exclusion was sometimes self‐imposed, with a sense of it being ‘the lesser of two evils’. Parents made decisions to proactively remove their children from mainstream activities because of concerns about unmet needs and inability to maintain safety and to prevent formal exclusion (Kiernan et al. 2019). Carers ‘withdrew’ from the community for self‐protection from peoples' hurtful reactions to their relative's behaviours and behaviour management strategies (Kiernan et al. 2019). Others avoided certain professionals due to ‘really awful’ relationships (Stock et al. 2024).

Experiences of isolation, discrimination and being unsupported likely explain why carers valued—and went onto develop—peer support groups (Kiernan et al. 2019), connecting them with others in similar positions.

##### 3.5.3.4 A Positive Shift

Some carers gained positives from their experiences and underwent transformative processes. They described personal growth, fostering their values and strengths and changed mindsets. Carers spoke of the ‘joy’ and ‘pride’ they experienced from their relatives, alongside re‐adjusting their views about what was important; ‘we're seeing real progress, she's able to put a toothbrush in her mouth’ (Gore, McGill, and Hastings 2019, 1708). Some felt they had become ‘more empathetic […] and patient with people’ (Chase and McGill 2019, 141).

During admissions, carers actively identifying their positive attributes provided reassurance in the face of role insecurity and vulnerability (James 2016). External validation from professionals was ‘refreshing and empowering’ (Gore, McGill, and Hastings 2019, 1708). Engaging in comparison to other carers they perceived to be less involved than them helped ‘substantiate their value and commitment’ (James 2016, 45). Authors reflected on the ‘fortitude and resilience’ of carers and the importance of harnessing their dedication to their loved one (Stock et al. 2024).

This recognition and development of their strengths and values inspired personal choices and ‘philanthropic’ action (Kiernan et al. 2019). Some used their resourcefulness to develop non‐traditional supports to meet the needs of their relatives and similar people (Kiernan et al. 2019). Others chose careers within the field due to a ‘passion to improve people's lives’ (Chase and McGill 2019, 141).

This seemed a protective mechanism; endeavouring to turn pain and hardships into something meaningful; ‘amongst all the bad things that have happened to us, I have a little niche’ (Gore, McGill, and Hastings 2019, 1706). It helped preserve a sense of self and value, as a carer and as an individual beyond their caring role (James 2016).

### 3.6 Line‐of‐Argument

The complexity of needs, emotional and relational experiences and required support was a theme running throughout the papers. The intersection of co‐occurring difficulties contributed to this, described by one parent as a ‘field of carnage’ (Botterill et al. 2019, 96). While there were shared aspects of caregivers' experiences, there were also idiosyncrasies with various BtC and mental health difficulties being discussed, including self‐harming, suicide attempts, anxiety, depression, violence and withdrawal.

Caregiving is multifaceted, including responsibilities of acquiring complex information, educating others, advocacy, protection and ‘battling’ with services, within the context of a lack of appropriate services and poor inter‐service collaboration. Marginalising attitudes and limited understanding, skills and expertise of professionals made experiences of services more burdensome than helpful. Carers wanted accessible points of contact; knowledgeable and consistent professionals; person‐centred, strengths‐based and contextually meaningful approaches and carer‐specific support and involvement in care. These experiences contributed to carers feeling powerless, undervalued, isolated, with wide‐ranging detrimental impacts on their sense of selves and wellbeing. Carers perceived their experience of caregiving as unique and different to ‘normal families’.

## 4 Discussion

This review synthesised qualitative literature exploring the experiences of caring for family members with intellectual disabilities displaying BtC and/or mental health difficulties. High levels of stress, poor physical and mental health, loneliness and burnout in carers of individuals with intellectual disabilities are well‐established (Cantwell et al. 2014; Goudie et al. 2014; Marquis et al. 2019). The intersection of needs places a higher burden on caregivers (Douma et al. 2006) and concomitantly results in services being withdrawn or restricted due to inflexible eligibility criteria and services being unable to safely meet the needs and manage risks (Griffith and Hastings 2014). This increased carers' feelings of alienation, placing them at greater risk of distress.

Despite the synthesised papers being published after ‘Building the Right Support’ (NHS England 2015), it was striking that care services continued to be more burdensome than helpful (Griffith and Hastings 2014) and even abusive in hospitals (Stock et al. 2024). Accessing appropriate support is a continued challenge for carers (Griffith and Hastings 2014; James 2013), leaving them balancing the need for advocacy, self‐preservation and complying with ‘bureaucratic’ systems to prevent further alienation and conflict. As reported previously, care was offered at ‘crisis’ point, requiring more restrictive interventions and hospitalisation, leading to greater distress for families (Weiss and Lunsky 2010; Wodehouse and McGill 2009).

Professionals often lacked expertise, held discriminatory beliefs and did not take carers' concerns seriously until other professionals advocated for them (James 2013). Inconsistent care resulted from poor inter‐agency collaboration and communication and between carers and services. Carers feeling dismissed, not listened to nor fully informed and excluded from their relatives' care is not novel (Faust and Scior 2008; McGill et al. 2006; Wodehouse and McGill 2009). Helpful experiences of care are sparse and inconsistent (Griffith and Hastings 2014). Carers valued and advocated for proactive and preventative models of care, having a key point of contact and consideration to be given to family contexts and goals (Faust and Scior 2008; Weiss and Lunsky 2010). Carers viewed themselves as experts of their relative and wanted meaningful partnership with professionals. However, it is imperative that the empowerment and involvement of carers does not come at the expense of diminishing or neglecting the voices of the individual with an intellectual disability. Family caregivers can act as ‘fierce defenders’ against the system to protect their family's dignity and rights, which can overpower adults with intellectual disabilities (Lashewicz et al. 2014). Thus, care should be holistic, respecting the views and needs of all stakeholders.

Caregivers highlighted significant detrimental impacts on their wellbeing. This resulted from unhelpful and harmful experiences of services, juggling caregiving responsibilities and seeing their relative's distress. Altered identities, increased psychological stress and poor mental health of carers have been discussed previously (Griffith and Hastings 2014; James 2013). Training is needed to help carers develop skills in mitigating and managing BtC and notice signs of mental health difficulties in their relative, alongside carer‐specific emotional and psychological support (James 2013).

This study reflects others that have reported carers generally and those of people with intellectual disabilities, experience personal growth, improved relationships, re‐evaluation of priorities and relatives being sources of happiness (Beighton and Wills 2019; Saragosa et al. 2022). Contrastingly to Griffith and Hastings (2014), no carers depicted their caregiving role as wholly fulfilling. There was little discussion of love underpinning relationships between carers and relatives (Griffith and Hastings 2014) and the elements of ‘joy’ depicted by Kearney and Griffin (2001) were under‐emphasised compared to the hardships. Carers experienced deficit‐driven care as disempowering and demotivating, wanting greater recognition of strengths and achievements. Problem‐saturated narratives have become dominant and may only represent one caregiving perspective. Nonetheless, positive aspects are ‘pervasive and diverse’ (Beighton and Wills 2019, 1273). Identifying positives can contribute to meaning‐making processes, enabling adaption to caregiving roles and experiences, which is key for lifelong caring (Beighton and Wills 2017) and preserving a sense of self. Greater focus on positive elements of caregiving, clinically and in research, may be beneficial for carers' wellbeing, generating hope and enabling them to maintain their roles. This should be done respectfully and thoughtfully, so as not to invalidate carers' significant concerns or struggles (Saragosa et al. 2022).

Carers' experiences of services were given greater weight in the research than experiences of their relatives' BtC or mental health difficulties. Perhaps carers' motivations for participating in research were about advocating for better services in a forum in which they perceived their concerns would be taken seriously. Carers may attribute the intensity and complexity of their own and their relatives' difficulties to poor services and the challenges navigating them (James 2013), possibly perceiving that they will persist without service improvement, so focused their efforts here.

It is possible carers felt unable or unwilling to discuss experiences of relatives' emotional and behavioural difficulties, especially when these were violent. Carers implied feelings of shame about their own emotions and views in relation to their relatives, avoiding discussing these with their personal support networks. They also reported perceived and real judgements and criticisms by others due to their relatives' behaviours. Isham et al. (2020) discussed the taboo nature of the topic and carers' apprehensions about stigmatising individuals with mental and physical health problems who act violently or unpredictably. These carers are a ‘hidden population’ and may underplay the impact of harm and nature of difficulties to avoid scrutiny, maintain a degree of control over who is involved in their relatives' lives and prevent relatives being removed from the home (Isham et al. 2021). Concealing these experiences may be another way carers protect their loved ones. However, higher secrecy coping is associated with depression and lower quality of life (Serchuk et al. 2021).

Nonetheless, there were hints at these experiences within the findings; anonymity within research interviews possibly increased disinhibition and enabled carers to open up. There is a positive association between self‐disclosure and anonymity (Clark‐Gordon et al. 2019). Limited opportunities to discuss these aspects of caregiving may exacerbate disempowerment, isolation and marginalisation, so understanding these experiences alongside carers' feelings of love, loyalty and responsibility towards family members is imperative (Isham et al. 2021).

### 4.1 Limitations

Across included studies, demographic data was poorly reported, restricting the conclusions that can be made. Of particular importance would be ethnic information, additional diagnoses and the living circumstances of family members with intellectual disabilities. Based on the reported data, family carers were predominantly female. While this is in keeping with the national statistics, with more than half of all unpaid carers in the United Kingdom being women (Office for National Statistics 2023), male carers' voices may be underrepresented. Only one paper specifically addressed sibling carers' experiences; this population continues to be underrepresented in both clinical and research arenas. The quality assessment revealed a widespread lack of consideration of relationships between researchers and participants, making it hard to determine the researchers' roles, potential biases and attempts to minimise the influence of these on the findings (Anney 2014).

While all papers were published after the national plan for reforming services (NHS England 2015), it was difficult to determine the dates of data collection in most studies and one explicitly collected data from inpatient settings between 2009 and 2013 (James 2016). Nonetheless, this allowed comparisons to be drawn with Stock et al. (2024), revealing consistencies in family carers' experiences of mental health hospitals, suggesting minimal change following Transforming Care.

Two papers utilising online surveys had large sample sizes, increasing the review's pooled sample size (Paulauskaite et al. 2021; Sheehan et al. 2018), however, contained limited qualitative data. Less data could be extracted from papers that included paid carers' views (Kouroupa, Hamza, et al. 2023; Kouroupa, Hassiotis, et al. 2023). Biases may have arisen from those with more difficult experiences participating in research, particularly, given the self‐selecting nature of participation in several papers. It is plausible this contributed to the limited discussion about perceived benefits and positive aspects of caregiving.

The review aimed to broadly capture experiences of caring for individuals with intellectual disabilities and mental health difficulties. Across the papers, there was limited information about formal mental health diagnoses, with mental health difficulties often described through symptoms (e.g., change in appetite, crying) or presumed due to being under mental health services. This may be indicative of broader issues prevalent for this population, including atypical presentations, impaired communication and mental health literacy and diagnostic overshadowing (Sheehan et al. 2015). No papers included people diagnosed with severe and enduring mental health difficulties (e.g., psychosis and eating disorders), despite some individuals with intellectual disabilities experiencing these (Mazza et al. 2020). Understanding carers' experiences of these unique co‐occurring needs is essential for ensuring access to and development of specialist support services.

### 4.2 Clinical Implications

Systemic models and care approaches are essential. Carers should be provided with information and training, be listened to, valued in their role and meaningfully involved with day‐to‐day care planning and interventions (Bigby et al. 2019; James 2013). Carers may benefit from professionals identifying their unique goals, needs and positive aspects of caring—adopting person‐centred and strengths‐based approaches, providing carer‐specific support and developing tailored and realistic plans (Beighton and Wills 2019). Transparency in service offers could mitigate against carers feeling disappointed and distrusting of services. Greater recognition of and opportunities for conversations about ‘taboo’ experiences of violent or harmful BtC may help safeguard and support carers' wellbeing (Isham et al. 2021). Developing peer support opportunities may increase carers' experiences of belonging and facilitate information sharing (Dew et al. 2019). Together, these could help prevent situations escalating to crisis point, in turn, reducing carer burnout and restrictive interventions and inappropriate admissions (Griffith and Hastings 2014).

Organisations are pivotal in empowering carers; however, professionals operate within the broader context of organisational constraints and may themselves need increased support and resources to be empowered and empowering (Petriwskyj et al. 2016). This paper has not focused on professionals' experiences and looking at multiple sources of evidence is key. In other healthcare settings, reflective practice can mitigate the impact of and reduce, occupational stress and burnout, which has the potential to reduce staff turnover (O'Neill et al. 2019), in turn, improving the quality and experiences of care. Thus, embedding reflective practices in services could be beneficial.

Enhanced partnership and signposting between services, including third sector organisations, may be advantageous (Durand et al. 2024), alongside an accessible point of contact to coordinate care (James 2013). All services should understand BtC for people with intellectual disabilities (Griffith and Hastings 2014; James 2013). Professionals, including those working in mainstream mental health services and respite provisions, might benefit from regular training to ensure knowledge and skills are maintained and updated, facilitating implementation of reasonable adjustments. Supervisors and team/service leads should facilitate training opportunities and integration of training into practice (Rose et al. 2012).

Consideration should be given to service commissioning models, mapping service provisions onto individual needs. Interventions that are longer term, proactive and based on holistic understandings of individuals and their families should be offered (Shankar et al. 2020). Increased access to and inclusion within local communities could proactively promote belonging and connection, supporting carers' psychosocial wellbeing.

### 4.3 Research Implications

Future research should highlight male and sibling caregivers and those caring for individuals with a broader range of mental health difficulties. Improved reporting of demographic information could clarify who participates in research and ensure the views of marginalised, disempowered and/or hidden populations are heard. Research focusing on ‘taboo’ topics of caring for individuals displaying aggressive and disruptive BtC could help to reduce carers' feelings of shame, powerlessness and isolation (Isham et al. 2021). Exploring professionals' views of supporting individuals with intellectual disabilities displaying BtC could inform practice.

## 5 Conclusions

This review highlighted that caring for family members with intellectual disabilities displaying BtC and/or mental health difficulties is a complex journey and multi‐faceted role significantly impacting on carers' wellbeing. Carers advocated for collaborative, proactive, person‐ and family‐ centred and strengths‐based services and interventions. Training and psychosocial support for families and care professionals is required. Research should consider sibling and male carers' experiences, alongside further exploration of more ‘taboo’ aspects of caring for this population and caring for individuals with more diverse mental health difficulties co‐occurring with intellectual disabilities.

## Funding

The authors have no funding to report.

## Ethics Statement

Ethical approval was not applicable for this study as this was a review paper.

## Conflicts of Interest

The authors declare no conflicts of interest.

## Supporting information


**Supporting Information:** Search terms.

## Data Availability

The data that support the findings of this study are available on request from the corresponding author. The data are not publicly available due to privacy or ethical restrictions.

## References

[jar70226-bib-0001] Adams, T. M. , and A. Jahoda . 2019. “Listening to Mothers: Experiences of Mental Health Support and Insights Into Adapting Therapy for People With Severe or Profound Intellectual Disabilities.” International Journal of Developmental Disabilities 65, no. 3: 135–142. 10.1080/20473869.2019.1609306. 34141334 PMC8115544

[jar70226-bib-0002] Anney, V. N. 2014. “Ensuring the Quality of the Findings of Qualitative Research: Looking at Trustworthiness Criteria.” Journal of Emerging Trends in Educational Research and Policy Studies (JETERAPS) 5, no. 2: 272–281.

[jar70226-bib-0004] Beighton, C. , and J. Wills . 2017. “Are Parents Identifying Positive Aspects to Parenting Their Child With an Intellectual Disability or Are They Just Coping? A Qualitative Exploration.” Journal of Intellectual Disabilities 21, no. 4: 325–345. 10.1111/jar.12617. 27352854 PMC5703033

[jar70226-bib-0005] Beighton, C. , and J. Wills . 2019. “How Parents Describe the Positive Aspects of Parenting Their Child Who Has Intellectual Disabilities: A Systematic Review and Narrative Synthesis.” Journal of Applied Research in Intellectual Disabilities 32, no. 5: 1255–1279. 10.1111/jar.12617. 31111640 PMC6852490

[jar70226-bib-0006] Bigby, C. , M. Whiteside , and J. Douglas . 2019. “Providing Support for Decision Making to Adults With Intellectual Disability: Perspectives of Family Members and Workers in Disability Support Services.” Journal of Intellectual & Developmental Disability 44, no. 4: 396–409. 10.3109/13668250.2017.1378873.

[jar70226-bib-0007] Boland, A. , R. Dickson , and G. Cherry . 2017. Doing a Systematic Review: A Student's Guide, 2nd ed. SAGE Publications Ltd.

[jar70226-bib-0008] Botterill, S. , S. Cottam , A. Fowke , and K. Theodore . 2019. “'It Put Control Back Onto My Family Situation': Family Experiences of Positive Behaviour Support.” Advances in Mental Health and Intellectual Disabilities 13, no. 3–4: 91–101. 10.1108/AMHID-11-2018-0049.

[jar70226-bib-0009] Butler, A. , H. Hall , and B. Copnell . 2016. “A Guide to Writing a Qualitative Systematic Review Protocol to Enhance Evidence‐Based Practice in Nursing and Health Care.” Worldviews on Evidence‐Based Nursing 13, no. 3: 241–249. 10.1111/wvn.12134. 26790142

[jar70226-bib-0010] Cantwell, J. , O. T. Muldoon , and S. Gallagher . 2014. “Social Support and Mastery Influence the Association Between Stress and Poor Physical Health in Parents Caring for Children With Developmental Disabilities.” Research in Developmental Disabilities 35, no. 9: 2215–2223. 10.1016/j.ridd.2014.05.012. 24927515

[jar70226-bib-0011] Chase, J. , and P. McGill . 2019. “The Sibling's Perspective: Experiences of Having a Sibling With a Learning Disability and Behaviour Described as Challenging.” Tizard Learning Disability Review 24, no. 3: 138–146. 10.1108/TLDR-11-2018-0032.

[jar70226-bib-0012] Clark‐Gordon, C. V. , N. D. Bowman , A. K. Goodboy , and A. Wright . 2019. “Anonymity and Online Self‐Disclosure: A Meta‐Analysis.” Communication Reports 32, no. 2: 98–111. 10.1080/08934215.2019.1607516.

[jar70226-bib-0013] Critical Appraisal Skills Programme . 2018. “CASP Qualitative Studies Checklist.” Accessed in April 2024. https://casp‐uk.net/casp‐tools‐checklists/qualitative‐studies‐checklist/.

[jar70226-bib-0071] Department of Health . 2012. Transforming Care: A National Response to Winterbourne View Hospital. Department of Health Review: Final report https://www.gov.uk/government/publications/transforming‐care‐a‐national‐response‐to‐winterbourne‐view‐hospital.

[jar70226-bib-0014] Dew, A. , S. Collings , L. Dowse , A. Meltzer , and L. Smith . 2019. “‘I Don't Feel Like I'm in This on My Own’: Peer Support for Mothers of Children With Intellectual Disability and Challenging Behaviour.” Journal of Intellectual Disabilities 23, no. 3: 344–358. 10.1177/1744629519843012. 31018752

[jar70226-bib-0015] Douma, J. C. , M. C. Dekker , and H. M. Koot . 2006. “Supporting Parents of Youths With Intellectual Disabilities and Psychopathology.” Journal of Intellectual Disability Research 50, no. 8: 570–581. 10.1111/j.1365-2788.2006.00825.x. 16867064

[jar70226-bib-0016] Durand, M. , R. Nathan , S. Holt , S. Nall‐Evans , and C. Woodrow . 2024. “Who Is at Risk? Adults With Intellectual Disability at Risk of Admission to Mental Health Inpatient Care.” Journal of Applied Research in Intellectual Disabilities 37, no. 3: e13210. 10.1111/jar.13210. 38382461

[jar70226-bib-0017] Emerson, E. , C. Hatton , J. Robertson , et al. 2012. People With Learning Disabilities in England 2011. Improving Health & Lives: Learning Disabilities Observatory. https://www.glh.org.uk/pdfs/PWLDAR2011.pdf.

[jar70226-bib-0018] Emerson, E. , C. Kiernan , A. Alborz , et al. 2001. “The Prevalence of Challenging Behaviors: A Total Population Study.” Research in Developmental Disabilities 22, no. 1: 77–93. 10.1016/S0891-4222(00)00061-5. 11263632

[jar70226-bib-0019] Faust, H. , and K. Scior . 2008. “Mental Health Problems in Young People With Intellectual Disabilities: The Impact on Parents.” Journal of Applied Research in Intellectual Disabilities 21, no. 5: 414–424. 10.1111/j.1468-3148.2007.00411.x.

[jar70226-bib-0020] France, E. F. , M. Cunningham , N. Ring , et al. 2019. “Improving Reporting of Meta‐Ethnography: The eMERGe Reporting Guidance.” BMC Medical Research Methodology 19: 1–13. 10.1186/s12874-018-0600-0. 30709371 PMC6359764

[jar70226-bib-0021] Fraser of Allander Institute . 2021. “Learning Disabilities and the Value of Unpaid Care.” Accessed September 21, 2023. https://fraserofallander.org/publications/learning‐disabilities‐and‐the‐value‐of‐unpaid‐care/.

[jar70226-bib-0022] Gore, N. J. , P. McGill , and R. P. Hastings . 2019. “Making It Meaningful: Caregiver Goal Selection in Positive Behavioral Support.” Journal of Child and Family Studies 28, no. 6: 1703–1712. 10.1007/s10826-019-01398-5.

[jar70226-bib-0072] Gore, N. J. , P. McGill , and R. P. Hastings . 2019. “Making it Meaningful: Caregivergoal Selection in Positive Behavioral Support.” Journal of Child and Family Studies 28, no. 6: 1703–1712. 10.1007/s10826-019-01398-5.

[jar70226-bib-0023] Goudie, A. , M.‐R. Narcisse , D. E. Hall , and D. Z. Kuo . 2014. “Financial and Psychological Stressors Associated With Caring for Children With Disability.” Families, Systems & Health 32, no. 3: 280–290. 10.1037/fsh0000027. PMC4315505 24707826

[jar70226-bib-0024] Griffith, G. M. , and R. P. Hastings . 2014. “‘He's Hard Work, but He's Worth It’ the Experience of Caregivers of Individuals With Intellectual Disabilities and Challenging Behaviour: A Meta‐Synthesis of Qualitative Research.” Journal of Applied Research in Intellectual Disabilities 27, no. 5: 401–419. 10.1111/jar.12073. 24105755

[jar70226-bib-0026] Hatton, C. 2017. “Living Arrangements of Adults With Learning Disabilities Across the UK.” Tizard Learning Disability Review 22, no. 1: 43–50. 10.1108/TLDR-11-2016-0040.

[jar70226-bib-0027] Hiller, A. J. , and D. F. Vears . 2016. “Reflexivity and the Clinician‐Researcher: Managing Participant Misconceptions.” Qualitative Research Journal 16, no. 1: 13–25. 10.1108/QRJ-11-2014-0065.

[jar70226-bib-0028] Hughes, J. , R. Roberts , J. Tarver , et al. 2024. “It Wasn't the Strategies on Their Own’: Exploring Caregivers' Experiences of Accessing Services in the Development of Interventions for Autistic People With Intellectual Disability.” Autism 28: 13623613231196084. 10.1177/13623613231196084. PMC11067391 37712611

[jar70226-bib-0029] Hughes‐McCormack, L. A. , E. Rydzewska , A. Henderson , C. MacIntyre , J. Rintoul , and S.‐A. Cooper . 2017. “Prevalence of Mental Health Conditions and Relationship With General Health in a Whole‐Country Population of People With Intellectual Disabilities Compared With the General Population.” BJPsych Open 3, no. 5: 243–248. 10.1192/bjpo.bp.117.005462. 29034100 PMC5620469

[jar70226-bib-0030] Isham, L. , C. Bradbury‐Jones , and A. Hewison . 2020. “Female Family Carers' Experiences of Violent, Abusive or Harmful Behaviour by the Older Person for Whom They Care: A Case of Epistemic Injustice?” Sociology of Health & Illness 42, no. 1: 80–94. 10.1111/1467-9566.12986. 31515820

[jar70226-bib-0031] Isham, L. , C. Bradbury‐Jones , and A. Hewison . 2021. “‘This Is Still All About Love’: Practitioners' Perspectives of Working With Family Carers Affected by the Harmful Behaviour of the Older Person for Whom They Care.” British Journal of Social Work 51, no. 8: 3190–3208. 10.1093/bjsw/bcaa129.

[jar70226-bib-0032] Jacobs, M. , L. M. Woolfson , and S. C. Hunter . 2016. “Attributions of Stability, Control and Responsibility: How Parents of Children With Intellectual Disabilities View Their Child's Problematic Behaviour and Its Causes.” Journal of Applied Research in Intellectual Disabilities 29, no. 1: 58–70. 10.1111/jar.12158. 25753834

[jar70226-bib-0033] James, I. A. , K. Reichelt , E. Moniz‐Cook , and K. Lee . 2020. “Challenging Behaviour in Dementia Care: A Novel Framework for Translating Knowledge to Practice.” Cognitive Behaviour Therapist 13: e43. 10.1017/S1754470X20000434.

[jar70226-bib-0034] James, N. 2013. “The Formal Support Experiences of Family Carers of People With an Intellectual Disability Who Also Display Challenging Behaviour and/or Mental Health Issues: What Do Carers Say?” Journal of Intellectual Disabilities 17, no. 1: 6–23. 10.1177/1744629512472610. 23325117

[jar70226-bib-0035] James, N. 2016. “Family Carers' Experience of the Need for Admission of Their Relative With an Intellectual Disability to an Assessment and Treatment Unit.” Journal of Intellectual Disabilities 20, no. 1: 34–54. 10.1177/1744629515592073. 26113061

[jar70226-bib-0036] Kearney, P. M. , and T. Griffin . 2001. “Between Joy and Sorrow: Being a Parent of a Child With Developmental Disability.” Journal of Advanced Nursing 34, no. 5: 582–592. 10.1046/j.1365-2648.2001.01787.x. 11380726

[jar70226-bib-0037] Kiernan, J. , D. Mitchell , J. Stansfield , and C. Taylor . 2019. “Mothers' Perspectives on the Lived Experience of Children With Intellectual Disability and Challenging Behaviour.” Journal of Intellectual Disabilities 23, no. 2: 175–189. 10.1177/1744629517737149. 29153009

[jar70226-bib-0038] Knapp, M. , A. Comas‐Herrera , J. Astin , J. Beecham , and C. Pendaries . 2005. “Intellectual Disability, Challenging Behaviour and Cost in Care Accommodation: What Are the Links?” Health & Social Care in the Community 13, no. 4: 297–306. 10.1111/j.1365-2524.2005.00539.x. 15969700

[jar70226-bib-0039] Kouroupa, A. , L. Hamza , A. Rafiq , et al. 2023. “Stakeholder Views on the Barriers and Facilitators of Psychosocial Interventions to Address Reduction in Aggressive Challenging Behaviour in Adults With Intellectual Disabilities.” NIHR Open Research 3: 40. 10.3310/nihropenres.13437.1. 37881460 PMC10593323

[jar70226-bib-0040] Kouroupa, A. , A. Hassiotis , L. Hamza , et al. 2023. “Stakeholder Perspectives on Intensive Support Teams for Adults With Intellectual Disabilities Who Display Behaviour That Challenges in England.” Journal of Applied Research in Intellectual Disabilities 36: 1101–1112. 10.1111/jar.13129. 37271584

[jar70226-bib-0041] Lashewicz, B. , J. Mitchell , E. Salami , and S. Cheuk . 2014. Understanding and Addressing Voices of Adults With Disabilities Within Their Family Caregiving Contexts: Implications for Capacity, Decision‐Making and Guardianship. Law Commission of Ontario. https://www.lco‐cdo.org/wp‐content/uploads/2014/01/capacity‐guardianship‐commissioned‐paper‐lashewicz.pdf.

[jar70226-bib-0042] Lunsky, Y. , A. Tint , S. Robinson , M. Gordeyko , and H. Ouellette‐Kuntz . 2014. “System‐Wide Information About Family Carers of Adults With Intellectual/Developmental Disabilities—A Scoping Review of the Literature.” Journal of Policy and Practice in Intellectual Disabilities 11, no. 1: 8–18. 10.1111/jppi.12068.

[jar70226-bib-0043] Marquis, S. , M. V. Hayes , and K. McGrail . 2019. “Factors Affecting the Health of Caregivers of Children Who Have an Intellectual/Developmental Disability.” Journal of Policy and Practice in Intellectual Disabilities 16, no. 3: 201–216. 10.1111/jppi.12283.

[jar70226-bib-0044] Maulik, P. K. , M. N. Mascarenhas , C. D. Mathers , T. Dua , and S. Saxena . 2011. “Prevalence of Intellectual Disability: A Meta‐Analysis of Population‐Based Studies.” Research in Developmental Disabilities 32, no. 2: 419–436. 10.1016/j.ridd.2010.12.018. 21236634

[jar70226-bib-0045] Mazza, M. G. , A. Rossetti , G. Crespi , and M. Clerici . 2020. “Prevalence of Co‐Occurring Psychiatric Disorders in Adults and Adolescents With Intellectual Disability: A Systematic Review and Meta‐Analysis.” Journal of Applied Research in Intellectual Disabilities 33, no. 2: 126–138. 10.1111/jar.12654. 31430018

[jar70226-bib-0046] McGill, P. , E. Papachristoforou , and V. Cooper . 2006. “Support for Family Carers of Children and Young People With Developmental Disabilities and Challenging Behaviour.” Child: Care, Health and Development 32, no. 2: 159–165. 10.1111/j.1365-2214.2006.00600.x. 16441850

[jar70226-bib-0047] McIntyre, L. L. , J. Blacher , and B. L. Baker . 2002. “Behaviour/Mental Health Problems in Young Adults With Intellectual Disability: The Impact on Families.” Journal of Intellectual Disability Research 46, no. 3: 239–249. 10.1046/j.1365-2788.2002.00371.x. 11896809

[jar70226-bib-0048] McKenzie, K. , C. Mayer , K. J. Whelan , A. McNall , S. Noone , and J. Chaplin . 2018. “The Views of Carers About Support for Their Family Member With an Intellectual Disability: With a Focus on Positive Behavioural Approaches.” Health & Social Care in the Community 26, no. 1: e56–e63. 10.1111/hsc.12475. 28695628

[jar70226-bib-0073] Mental Health Act 2007. (2007). UK Government.

[jar70226-bib-0049] NHS England . 2015. “Building the Right Support: A National Plan to Develop Community Services and Close Inpatient Facilities for People With a Learning Disability and/or Autism Who Display Behaviour That Challenges, Including Those With a Mental Health Condition.” April 11, 2022. https://www.england.nhs.uk/wp‐content/uploads/2015/10/ld‐nat‐imp‐plan‐oct15.pdf.

[jar70226-bib-0050] NHS England . 2017. “Transforming Care. Model Service Specifications: Supporting Implementation of the Service Model.” Accessed September 2023. https://www.england.nhs.uk/wp‐content/uploads/2017/02/model‐service‐spec‐2017.pdf.

[jar70226-bib-0051] Noblit, G. W. , and R. D. Hare . 1988. Meta‐Ethnography: Synthesizing Qualitative Studies. Vol. 11. sage. 10.4135/9781412985000.

[jar70226-bib-0052] Office for National Statistics . 2023. “Unpaid Care, England Wales: Census 2021.” Accessed May 2024. https://www.ons.gov.uk/peoplepopulationandcommunity/healthandsocialcare/healthandwellbeing/bulletins/unpaidcareenglandandwales/census2021.

[jar70226-bib-0053] O'Neill, L. , J. Johnson , and R. Mandela . 2019. “Reflective Practice Groups: Are They Useful for Liaison Psychiatry Nurses Working Within the Emergency Department?” Archives of Psychiatric Nursing 33, no. 1: 85–92. 10.1016/j.apnu.2018.11.003. 30663630

[jar70226-bib-0054] Page, M. J. , J. E. McKenzie , P. M. Bossuyt , et al. 2021. “The PRISMA 2020 Statement: An Updated Guideline for Reporting Systematic Reviews.” BMJ 372: n71. 10.1136/bmj.n71. 33782057 PMC8005924

[jar70226-bib-0055] Paulauskaite, L. , O. Farris , H. M. Spencer , and A. Hassiotis . 2021. “My Son Can't Socially Distance or Wear a Mask: How Families of Preschool Children With Severe Developmental Delays and Challenging Behavior Experienced the COVID‐19 Pandemic.” Journal of Mental Health Research in Intellectual Disabilities 14, no. 2: 225–236. 10.1080/19315864.2021.1874578.

[jar70226-bib-0056] Petriwskyj, A. , J. Franz , and B. Adkins . 2016. “Parents, Services and System: An Exploration of Power Dynamics in Future Planning Among Parent Carers for People With Disability.” Disability & Society 31, no. 8: 1081–1097. 10.1080/09687599.2016.1234367.

[jar70226-bib-0057] Pruijssers, A. , B. Van Meijel , M. Maaskant , W. Nijssen , and T. van Achterberg . 2014. “The Relationship Between Challenging Behaviour and Anxiety in Adults With Intellectual Disabilities: A Literature Review.” Journal of Intellectual Disability Research 58, no. 2: 162–171. 10.1111/jir.12012. 23336582

[jar70226-bib-0058] Rose, N. , J. Rose , and S. Kent . 2012. “Staff Training in Intellectual Disability Services: A Review of the Literature and Implications for Mental Health Services Provided to Individuals With Intellectual Disability.” International Journal of Developmental Disabilities 58, no. 1: 24–39. 10.1179/2047387711Y.0000000005.

[jar70226-bib-0059] Ross, H. , and N. Dodds . 2023. “Exploring Risk Factors for Admission to Children's Learning Disability Hospitals Using Interpretative Phenomenological Analysis.” British Journal of Learning Disabilities 51: 283–295. 10.1111/bld.12437.

[jar70226-bib-0060] Saragosa, M. , M. Frew , S. Hahn‐Goldberg , A. Orchanian‐Cheff , H. Abrams , and K. Okrainec . 2022. “The Young Carers' Journey: A Systematic Review and Meta Ethnography.” International Journal of Environmental Research and Public Health 19, no. 10: 5826. 10.3390/ijerph19105826. 35627362 PMC9140828

[jar70226-bib-0061] Sattar, R. , R. Lawton , M. Panagioti , and J. Johnson . 2021. “Meta‐Ethnography in Healthcare Research: A Guide to Using a Meta‐Ethnographic Approach for Literature Synthesis.” BMC Health Services Research 21: 1–13. 33419430 10.1186/s12913-020-06049-w PMC7796630

[jar70226-bib-0062] Serchuk, M. D. , P. W. Corrigan , S. Reed , and J. L. Ohan . 2021. “Vicarious Stigma and Self‐Stigma Experienced by Parents of Children With Mental Health and/or Neurodevelopmental Disorders.” Community Mental Health Journal 57, no. 8: 1537–1546. 10.1007/s10597-021-00774-0. 33475886 PMC8531051

[jar70226-bib-0063] Shankar, R. , F. Haut , and K. Courtenay . 2020. Mental Health Services for Adults With Mild Intellectual Disability, Royal College of Psychiatrists. https://pearl.plymouth.ac.uk/pms‐research/1087.

[jar70226-bib-0064] Sheehan, R. , A. Hassiotis , K. Walters , D. Osborn , A. Strydom , and L. Horsfall . 2015. “Mental Illness, Challenging Behaviour, and Psychotropic Drug Prescribing in People With Intellectual Disability: UK Population Based Cohort Study.” BMJ 351: h4326. 10.1136/bmj.h4326. 26330451 PMC4556752

[jar70226-bib-0065] Sheehan, R. , K. Kimona , A. Giles , V. Cooper , and A. Hassiotis . 2018. “Findings From an Online Survey of Family Carer Experience of the Management of Challenging Behaviour in People With Intellectual Disabilities, With a Focus on the Use of Psychotropic Medication.” British Journal of Learning Disabilities 46, no. 2: 82–91. 10.1111/bld.12216.

[jar70226-bib-0066] Siddaway, A. P. , A. M. Wood , and L. V. Hedges . 2019. “How to Do a Systematic Review: A Best Practice Guide for Conducting and Reporting Narrative Reviews, Meta‐Analyses, and Meta‐Syntheses.” Annual Review of Psychology 70: 747–770. 10.1146/annurev-psych-010418-102803. 30089228

[jar70226-bib-0067] Stock, M. , M. Mulholland , V. Cooper , et al. 2024. “‘The Whole Thing Is Beyond Stress’: Family Perspectives on the Experience of Hospitalisation Through to Discharge for Individuals With Severe Learning Disabilities and Complex Needs.” British Journal of Learning Disabilities 52: 633–643. 10.1111/bld.12595.

[jar70226-bib-0068] Venville, A. , A.‐M. Sawyer , M. Long , N. Edwards , and S. Hair . 2015. “Supporting People With an Intellectual Disability and Mental Health Problems: A Scoping Review of What They Say About Service Provision.” Journal of Mental Health Research in Intellectual Disabilities 8, no. 3–4: 186–212. 10.1080/19315864.2015.1069912.

[jar70226-bib-0069] Weiss, J. , and Y. Lunsky . 2010. “Service Utilization Patterns in Parents of Youth and Adults With Intellectual Disability Who Experienced Behavioral Crisis.” Journal of Mental Health Research in Intellectual Disabilities 3, no. 3: 145–163. 10.1080/19315864.2010.490617.

[jar70226-bib-0070] Wodehouse, G. , and P. McGill . 2009. “Support for Family Carers of Children and Young People With Developmental Disabilities and Challenging Behaviour: What Stops It Being Helpful?” Journal of Intellectual Disability Research 53, no. 7: 644–653. 10.1111/j.1365-2788.2009.01163.x. 19298502

