# Supplementary material for: Exploring the Experiences of Caring for a Family Member With Intellectual Disabilities Displaying Behaviours That Challenge and/or Mental Health Difficulties Within the United Kingdom: A Meta‐Ethnographic Review
Source: J Appl Res Intellect Disabil. 2026 Apr 17;39(2):e70226. doi: 10.1111/jar.70226 (PMC13088942; doi:10.1111/jar.70226)
Supplement: Supplementary file 1 — Supporting Information: Search terms. [file JAR-39-e70226-s001.doc]

##### Supplementary Material: Search Terms

| **Concept 1** | **Concept 2** | **Concept 3** | **Concept 4** | **Concept 5** |
| --- | --- | --- | --- | --- |
| Mental health | Challeng* behaviour* | Parent* | Intellectual disab* | Experience* |
| Mental disorder* | Complex behaviour* | Sibling* | Intellectual impair* | Perspective* |
| Mental condition* | Behaviour* of concern* | Family* | Cognitive impair* | Attitude* |
| Mental problem* | Behaviour* that challenge* | Family car* | Learning disab* | View* |
| Mental ill-health* | Problem behaviour* | Mother* | Learning disorder* |  |
| Ill-mental health | Behaviour problem* | Father* | Developmental disab* |  |
| Mental instabilit* | Disruptive behaviour* | Maternal | Developmental delay* |  |
| Psychological* distress* | Destructive behaviour* | Paternal | Global delay* |  |
| Psychiatric* | Abberant behaviour* | Informal car* | Mental* handicap* |  |
| Emotion* distress* | Self-injur* | Carer* | Mental* impair* |  |
| Depress* | Self-harm* | Caregiv* | Mental* retard* |  |
| Anxi* | Self-stimulat* | Care-giv* | Mental* disab* |  |
| Mood disorder* | Aggressi* |  |  |  |
| Bipolar | Anger |  |  |  |
| Emotion* disorder* | Violen* |  |  |  |
| Psychosis | Destruction of property |  |  |  |
| Psychotic disorder* | Stereotyp* behaviour* |  |  |  |
| Personality disorder* |  |  |  |  |
| Psychosomatic disorder* |  |  |  |  |
| Psychopatholog* |  |  |  |  |
| Paranoi* |  |  |  |  |
| Stress |  |  |  |  |
| Neurosis |  |  |  |  |
